# Species Diversity, Distribution, and Phylogeny of *Exophiala* with the Addition of Four New Species from Thailand

**DOI:** 10.3390/jof8080766

**Published:** 2022-07-24

**Authors:** Tanapol Thitla, Jaturong Kumla, Surapong Khuna, Saisamorn Lumyong, Nakarin Suwannarach

**Affiliations:** 1 Master of Science Program in Applied Microbiology (International Program), Faculty of Science, Chiang Mai University, Chiang Mai 50200, Thailand; tanapol.thitla@gmail.com; 2 Department of Biology, Faculty of Science, Chiang Mai University, Chiang Mai 50200, Thailand; jaturong_yai@hotmail.com (J.K.); trio_9@hotmail.com (S.K.); 3 Research Center of Microbial Diversity and Sustainable Utilization, Chiang Mai University, Chiang Mai 50200, Thailand; 4 Academy of Science, The Royal Society of Thailand, Bangkok 10300, Thailand

**Keywords:** black yeast-like fungi, *Exophiala*, phylogeny, polymorphic fungi, taxonomy

## Abstract

The genus *Exophiala* is an anamorphic ascomycete fungus in the family *Herpotrichiellaceae* of the order *Chaetothyriales*. *Exophiala* species have been classified as polymorphic black yeast-like fungi. Prior to this study, 63 species had been validated, published, and accepted into this genus. *Exophiala* species are known to be distributed worldwide and have been isolated in various habitats around the world. Several *Exophiala* species have been identified as potential agents of human and animal mycoses. However, in some studies, *Exophiala* species have been used in agriculture and biotechnological applications. Here, we provide a brief review of the diversity, distribution, and taxonomy of *Exophiala* through an overview of the recently published literature. Moreover, four new *Exophiala* species were isolated from rocks that were collected from natural forests located in northern Thailand. Herein, we introduce these species as *E. lamphunensis*, *E. lapidea*, *E. saxicola*, and *E. siamensis*. The identification of these species was based on a combination of morphological characteristics and molecular analyses. Multi-gene phylogenetic analyses of a combination of the internal transcribed spacer (ITS) and small subunit (nrSSU) of ribosomal DNA, along with the translation elongation factor (*tef*), partial β-tubulin (*tub*), and actin (*act*) genes support that these four new species are distinct from previously known species of *Exophiala*. A full description, illustrations, and a phylogenetic tree showing the position of four new species are provided.

## 1. Introduction

**Figure 1:**
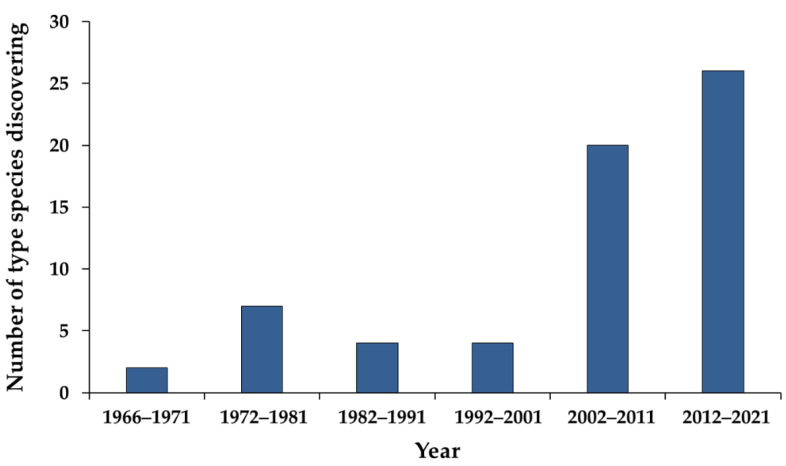
The discovery of *Exophiala*-type species since 1966 to the present time.

The genus *Exophiala* was initially described by Carmichael [1] in 1966 with *Exophiala salmonis* as the species type. *Exophiala* species are anamorphic ascomycete fungi belonging to the family *Herpotrichiellaceae* of the order *Chaetothyriales* [2]. The teleomorphic state of *Exophiala* has been classified in the genus *Capronia* [3,4]. *Exophiala* species are commonly known as black yeast-like fungi that are mainly characterized by annellidic conidiogenesis and yeast-like states [3,5,6]. However, several studies have indicated that *Exophiala* species are polymorphic fungi according to certain morphological variations that include budding cells, phialidic, catenate, or sympodial synanamorphs [3,7,8,9]. Due to the wide range of morphological variations, it is difficult to identify *Exophiala* by their morphological characteristics alone [6,10]. Frequently, when only morphological characteristics have been used to identify specimens within the *Exophiala* species, they can often be misidentified. Some species of *Exophiala* have been identified in the following genera: *Graphium*, *Haplographium*, *Hormiscium*, *Phaeococcomyces*, *Phaeococcus*, *Phialophora*, *Pullularia*, *Sarcinomyces*, *Sporocybe*, *Trichosporum*, and *Torula* [3,11,12,13,14,15,16,17]. Therefore, it is essential to identify *Exophiala* species by applying a molecular approach. Ribosomal DNA (ITS and nrSSU regions) and protein-coding (*tef*, *tub*, and *act*) genes have provided researchers with a powerful tool in the identification of the *Exophiala* species [3,4,5,7,11,17,18]. Currently, a combination of morphological characterization and multi-gene molecular phylogeny are being used for the accurate identification of the *Exophiala* species. From 1966 to the present, a total of 63 *Exophiala* species have been accepted and recorded in the Index Fungorum [19] and previous reports [4,20]. It has been revealed that the highest number of type species of the *Exophiala* were discovered during the period from 2012 to 2021 (26 species), followed by the periods from 2002 to 2011 (20 species) and 1972 to 1981 (7 species) (Figure 1). It can be expected that the trend of the discovery of new *Exophiala* species increasing will continue in the future.

**Figure 2:**
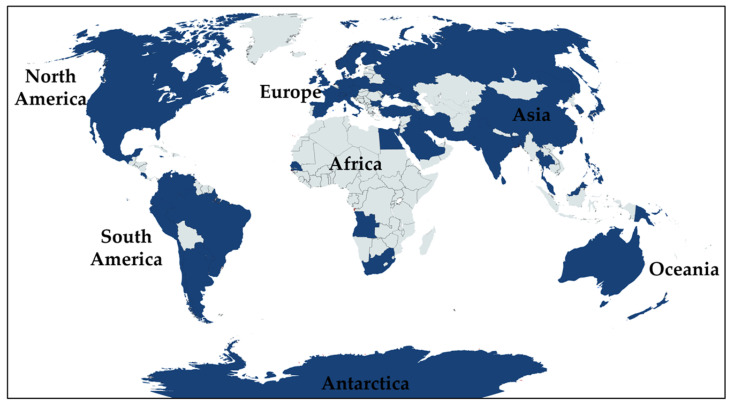
Global distribution of *Exophiala* species. Area and countries where *Exophiala* species have been discovered are indicated in dark blue color.

*Exophiala* species have been successfully isolated in various habitats worldwide. This would indicate their capacity to adapt to different ecosystems as summarized in Table 1. Several species have been found in various natural environments [3,18,21,22,23,24,25,26]. Some species have been isolated from anthropogenic places and objects (e.g., bathrooms, gasoline tanks of cars, washing machines, and kitchen sponges) [7,27,28,29]. Moreover, some of these species have been isolated from diseased humans and animals [1,3,8,20,30,31]. Consequently, *Exophiala* species are known to be widely distributed in tropical, subtropical, temperate, and polar areas throughout the world (Figure 2). According to the outcomes of previous studies, it has been reported that the highest number of *Exophiala* species were found in Europe, accounting for 35 species. This is followed by North America (25 species), Asia (24 species), South America (18 species), the Oceania region (11 species), Africa (7 species), and Antarctica (2 species). Of these, only *E*. *spinifera* has been found to be distributed across the world [6,32,33,34,35,36,37,38,39,40,41,42,43,44]. Moreover, *E*. *dermatitidis* has been discovered in regions throughout the world, with the exception of Antarctica and the Oceania region [7,15,16,31,32,45,46,47,48,49,50,51,52,53]. Accordingly, *E*. *jeanselmei* has been found in Asia, Europe, North America, the Oceania region, and South America [6,17,27,31,32,33,34,54,55,56,57,58,59,60,61,62,63,64,65,66,67]. However, nine species (*E. arunalokei*, *E*. *asiatica*, *E*. *calicioides*, *E*. *cinerea*, *E*. *clavispora*, *E*. *ellipsoidea*, *E*. *hongkongensis*, *E*. *nagquensis*, and *E*. *pseudooligosperma*) have been recorded only in Asia [4,18,20,30,31,32,68]. Thirteen species, namely *E*. *abietophila*, *E*. *bonariae*, *E*. *campbellii*, *E*. *italica*, *E*. *lacus*, *E*. *lavatrina*, *E*. *lignicola*, *E*. *mansonii*, *E*. *nidicola*, *E*. *psychrophila*, *E*. *quercina*, *E*. *radicis*, and *E*. *sideris*, have been discovered in Europe [3,5,7,9,15,21,24,69,70,71,72,73]. However, *E*. *xenobiotica* has not been reported in Africa [6,7,27,31,34,57,74].

**Figure 3:**
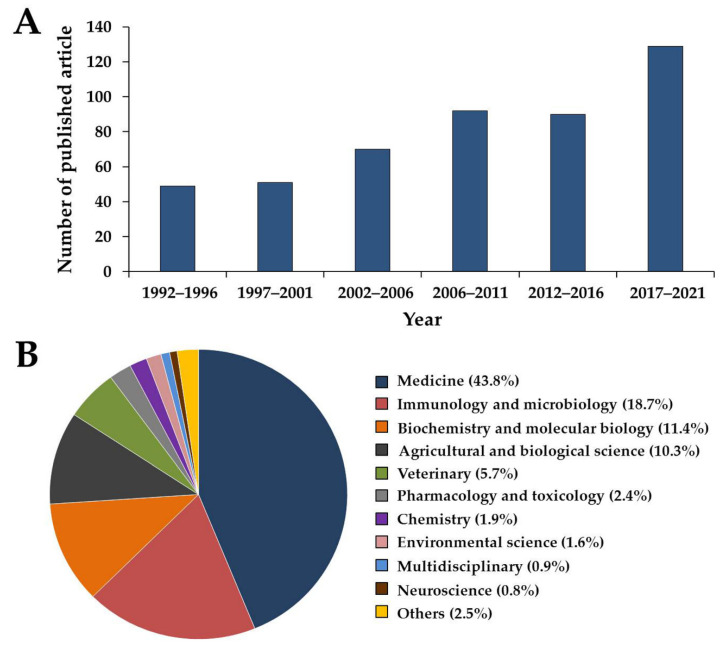
Number of research articles (**A**) and related field areas (**B**) between 1992 and 2021 with “*Exophiala*” as a keyword. The search was performed using the Scopus database (accessed on the 9 May 2022).

A search involving the keyword “*Exophiala*” retrieved 481 titles of research articles that had been published over the last 30 years (1992 to 2021) in the Scopus database [112]. The current upward trend associated with the research of *Exophiala* is expected to continue in the future (Figure 3A). It has been determined that the majority of applications for *Exophiala* have been reported in the medical field, accounting for 43.8%, followed by the fields of immunology and microbiology (18.7%), biochemistry and molecular biology (11.4%), agricultural and biological science (10.3%), veterinary medicine (5.7%), and pharmacology and toxicology (2.4%) (Figure 3B). 

There are 26 *Exophiala* species (41.3%) that have been reported as causal agents of human diseases [1,2,3,4,5,6,7,8,9,10,11,12,13,14,15,16,17,18,19,20,21,22,23,24,25,26,27,28,29,30,31,32,33,34,35,36,37,38,39,40,41,42,43,44,45,46,47,48,49,50,51,52,53,54,55,56,57,58,59,60,61,62,63,64,65,66,67,68,69,70,71,72,73,74,75,76,77,78,79,80,81,82,83,84,85,86,87,88,89,90,91,92,93,94,95,96,97,98,99,100,101,102,103,104,105,106,107,108,109,110,111]. In addition, seven species of *Exophiala* (11.1%), namely *E. angulospora*, *E*. *aquamarina*, *E. cancerae*, *E. equina*, *E. pisciphila*, *E. psychrophila*, and *E*. *salmonis*, were identified as pathogens of sea creatures. However, the remaining 34 *Exophiala* species (54.0%) have not been associated with pathogenicity in humans or animals [1,2,3,4,5,6,7,8,9,10,11,12,13,14,15,16,17,18,19,20,21,22,23,24,25,26,27,28,29,30,31,32,33,34,35,36,37,38,39,40,41,42,43,44,45,46,47,48,49,50,51,52,53,54,55,56,57,58,59,60,61,62,63,64,65,66,67,68,69,70,71,72,73,74,75,76,77,78,79,80,81,82,83,84,85,86,87,88,89,90,91,92,93,94,95,96,97,98,99,100,101,102,103,104,105,106,107,108,109,110,111] (Table 1). However, in some previous studies, some *Exophiala* species have been effectively used in agricultural and biotechnological applications. Examples of these include *E*. *pisciphila*, which was able to promote the plant growth of maize by increasing phosphorus absorption, photosynthesis, and tolerance of cadmium [113,114]. Furthermore, by effectively suppressing Fusarium-wilt disease in strawberries, *E*. *pisciphila* could be considered a biocontrol agent [109]. In terms of drug discovery, exophillic acid and its derivative compounds derived from *Exophiala* species have exhibited activity against HIV-1 integrase [115,116]. Importantly, the antimicrobial property of chlorohydroaspyrones and exophilin A produced from *Exophiala* species has been reported [117,118]. Interestingly, *Exophiala* has demonstrated the ability to degrade hydrocarbons (e.g., benzene, toluene, and xylene) that can be employed in bioremediation applications [25,119]. Although *Exophiala* species have been researched in a variety of applications, certain risks still remain. Therefore, further research should be conducted in the future, particularly with regard to the aspects of management and safety.

Currently, only three *Exophiala* species have been identified in Thailand, namely *E. dermatitidis*, *E. jeanselmei*, and *E. spinifera* [43,46,67]. Accordingly, many studies have proposed that Thailand has proven to be a hot spot for novel microfungal species discovery [120,121,122]. During investigations of rock-inhabiting fungi in northern Thailand during the period of 2020 to 2021, we obtained fifteen *Exophiala* strains that are potentially representative of new species. In the present study, we describe four new species, namely *E. lamphunensis*, *E. lapidea*, *E. saxicola*, and *E. siamensis*. These four new species were identified based on morphological and molecular data. To confirm their taxonomic status, phylogenetic relationships were determined by analysis of the combined sequence dataset of ITS, nrSSU, *tef*, *tub*, and *act* genes.

## 2. Materials and Methods

### 2.1. Sample Collection and Fungal Isolation

Rock samples were collected from four natural forests located in Lamphun (three sites; 18°32′11″ N 99°07′29″ E, 18°32′10″ N 99°07′30″ E, and 18°32′11″ N 99°07′30″ E) and Sukhothai (17°32′58″ N 99°29′49″ E) Provinces, northern Thailand. The samples comprising flourishing black colonies were collected with a sterile chisel, kept in plastic bags, and carried to the laboratory in an ice box. All collected rock samples were processed for the isolation of fungi immediately after reaching the laboratory. Fungi were isolated using the method described by Selbmann et al. [123] with some modifications. Rock samples were washed in 1% sodium hypochlorite for 10 min and rinsed 5 times in sterile water. Fungal isolation was performed by pulverizing the rock samples and sprinkling rock powder onto 2% malt extract agar (MEA; Difco, Le Pont de Claix, France) and dichloran-rose bengal agar (DRBC; Difco, Le Pont de Claix, France) supplemented with chloramphenicol 100 ppm. Plates were incubated at 25 **°C** for 4 weeks. Plates were then inspected every day. Fungal colonies with dark pigments were transferred to fresh MEA. Pure fungal strains were kept in 20% glycerol and deposited in the Culture Collection of Sustainable Development of Biological Resources Laboratory (SDBR), Faculty of Science, Chiang Mai University, Chiang Mai, Thailand.

### 2.2. Morphological and Growth Observations

Agar plugs (5 mm in diameter) from the edges of each fungal strain were transferred onto plates containing potato dextrose agar (PDA; Condalab, Madrid, Spain), MEA, and oatmeal agar (OA; Difco, Le Pont de Claix, France) and then kept at 25 °C in the dark. After four weeks of incubation, relevant colony features, including aerial mycelium and pigment production, were recorded and the colony diameter was measured. Cardinal growth temperatures were studied on MEA for 4 weeks in the dark at 4, 10, 15, 20, 25, 28, 30, 35, 37, and 40 °C using the method described by de Hoog et al. [3] with some modifications. A light microscope (Nikon Eclipse Ni-U, Tokyo, Japan) was used to study the micromorphological features of each fungal strain. The anatomical structure related to size data (e.g., hyphae, budding cells, conidia, and chlamydospore) was based on at least 50 measurements of each structure using the Tarosoft (R) Image.

### 2.3. DNA Extraction, Amplification, and Sequencing

A Fungal DNA Extraction Kit (FAVORGEN, Ping-Tung, Taiwan) was used to extract genomic DNA from the 3-week-old fungal culture of each strain that grew on MEA at 25 °C. Ribosomal DNA (ITS and nrSSU regions) and protein-coding (*tef*, *tub*, and *act*) genes were amplified by polymerase chain reaction (PCR) using suitable primers (Table 2). PCR amplifications were performed using 20-µL reaction mixtures containing 1 µL of genomic DNA, 1 µL of 10 µM forward and reverse primers, 10 µL of Quick TaqTM HS DyeMix (TOYOBO, Osaka, Japan), and 7 µL of deionized water. PCR amplification conditions consisted of an initial denaturation step conducted at 95 °C for 5 min, followed by 35 cycles of denaturation at 95 °C for 30 s, an annealing step for 30 s, at appropriate temperatures (Table 2), and an elongation step at 72 °C for 1 min on a peqSTAR thermal cycler (PEQLAB Ltd., Fareham, UK). PCR products were checked on 1% agarose gel electrophoresis and were purified using a PCR clean up Gel Extraction NucleoSpin^®^ Gel and a PCR Clean-up Kit (Macherey-Nagel, Düren, Germany). Purified PCR products were then sequenced by 1st Base Company (Kembangan, Malaysia).

### 2.4. Sequence Alignment 

The resulting ITS, nrSSU, *tef*, *tub*, and *act* sequences were assessed for similarity analysis in the GenBank database via BLAST searching. The sequences from this study, and those of closely related fungi, were obtained from the nucleotide GenBank database and previous studies as listed in Table 3. Multiple sequence alignment was carried out using MUSCLE in MEGA v. 6 [128] and the results were enhanced, when necessary, using BioEdit v.6.0.7 [129].

### 2.5. Phylogenetic Analyses

Phylogenetic analyses were performed using combination datasets of ITS, nrSSU, *tef*, *tub*, and *act* genes. *Cyphellophora eucalypti* CBS 124764 and *C. fusarioides* MUCL 44033 were used as the outgroup. Maximum likelihood (ML) and Bayesian inference (BI) methods were used to generate a phylogenetic tree. For ML analysis, 25 categories and 1000 bootstrap (BS) replications under the GTRCAT model [145] were performed on RAxML-HPC2 version 8.2.12 [146] on the CIPRES web portal [147]. The evolutionary model of nucleotide substitution for BI analysis was selected independently for each gene using MrModeltest v. 2.1 [148]. The GTR + I + G substitution model was the best fit for the ITS and nrSSU genes while the HKY + I + G substitution model was the best fit for the *tef* and *tub* genes, and the HKY + G substitution model was the best fit for the *act* gene. MrBayes v.3.2.6 was used for BI analysis [149]. In total, 6 simultaneous Markov chains were run for 5 million generations with random initial trees, wherein every 1000 generations were sampled. A burn-in phase was used to eliminate the first 2000 trees while the remaining trees were utilized to create a phylogram with a 50% majority-rule consensus. The Bayesian posterior probability (PP) was then calculated. Branches with BS and PP values of more than or equal to 70% and 0.95, respectively, were deemed to have been substantially supported. The tree topologies were visualized in FigTree v1.4.0 [150].

## 3. Results

### 3.1. Fungal Isolation and Morphological Observations

A total of fifteen fungal strains were obtained in this study. Thirteen strains were isolated from rock samples collected from Lamphun Province and two strains were isolated from rock samples collected from Sukhothai Province. All fungal strains were cultivated on MEA at various temperatures (4–40 °C) and the diameter of the colonies was measured after 4 weeks of incubation. The results indicated that temperature had a significant effect on fungal growth. The average colony diameter of each fungal strain is shown in Table 4. It was found that that all fungal strains could not grow at 4 and 40 °C. However, all fungal strains grew well in temperatures ranging from 25–30 °C, with the exception of the strains SDBR-CMU417 and SDBR-CMU418. Five fungal strains (SDBR-CMU404, SDBR-CMU405, SDBR-CMU406, SDBR-CMU407, and SDBR-CMU408) showed the highest average value of the colony diameter at 28 °C while eight fungal strains (SDBR-CMU409, SDBR-CMU410, SDBR-CMU411, SDBR-CMU412, SDBR-CMU413, SDBR-CMU414, SDBR-CMU415, and SDBR-CMU416) showed the highest average value of the colony diameter at 30 °C. The results indicate that the highest average value of the colony diameter of two fungal strains, namely SDBR-CMU417 and SDBR-CMU418, was found at 20 °C; however, they did not grow at 35 and 37 °C. Based on the morphological characteristics, all fungal isolates were initially identified as belonging to the genus *Exophiala*. The identification was then further confirmed by the multi-gene phylogenetic analysis of the ITS, nrSSU, *tub*, *tef*, and *act* sequences. 

### 3.2. Phylogenetic Results

**Figure 4:**
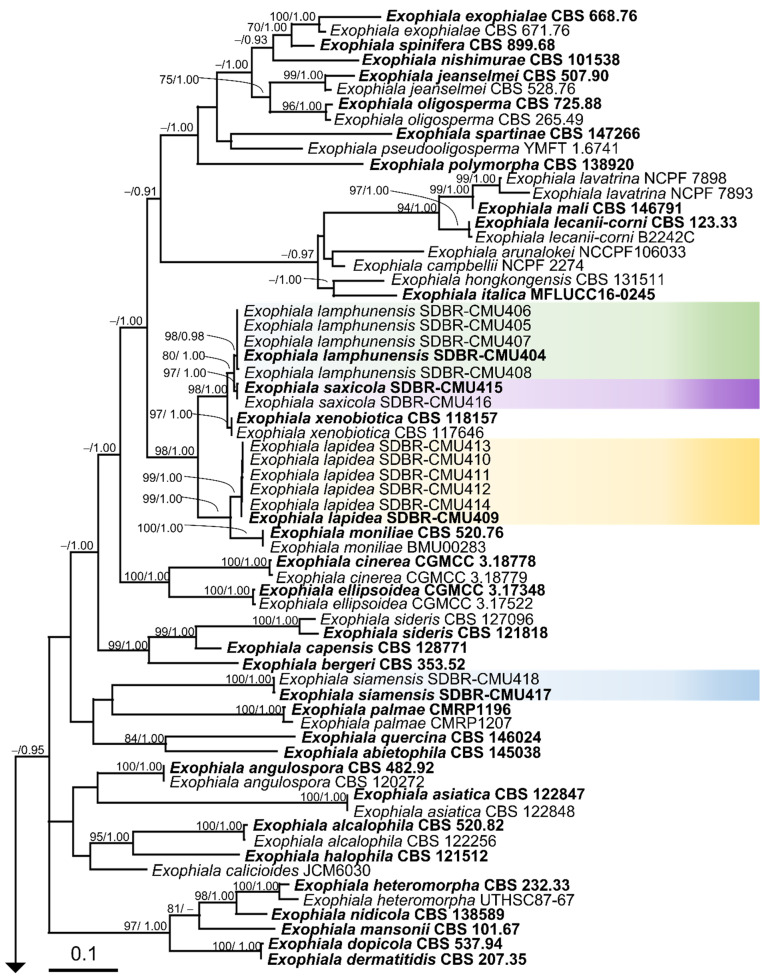

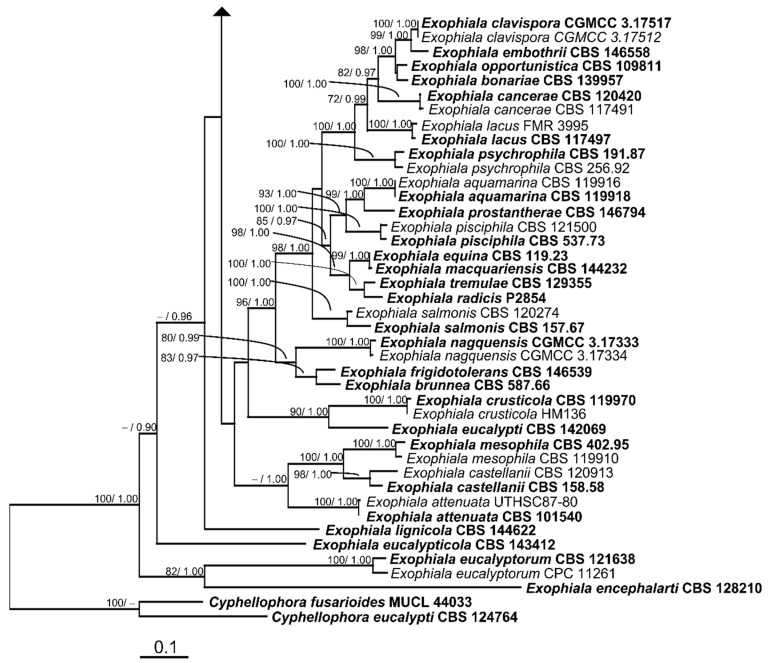
Phylogram generated from maximum likelihood analysis of 105 specimens of the combined ITS, nrSSU, *tub*, *tef*, and *act* genes. *Cyphellophora fusarioides* MUCL 44033 and *C. eucalypti* CBS 124764 were used as the outgroup. The numbers above branches show bootstrap percentages (left) and Bayesian posterior probabilities (right). Bootstrap values ≥ 70% and Bayesian posterior probabilities ≥ 0.95 are shown. The scale bar reflects the estimated number of nucleotide substitutions per site. Color bands represent the sequences of fungal species obtained in this study. Type species are in bold.

A phylogenetic tree was constructed using a combination of the ITS, nrSSU, *tub*, *tef*, and *act* genes containing 3616 characters, including gaps (ITS: 1–739, nrSSU: 740–1829, *tef*: 1830–2454, *tub*: 2455–3045, and *act*: 3046–3616). The phylogram was constructed, consisting of 105 specimens of *Exophiala* and 2 specimens of the outgroup (*Cyphellophora fusarioides* MUCL 44033 and *C. eucalypti* CBS 124764). RAxML analysis of the combined dataset yielded the best scoring tree, with a final log likelihood value of −38,143.750648. The matrix was comprised of 1880 distinct alignment patterns with 53.45% undetermined characters or gaps. Estimated base frequencies were recorded as follows: A = 0.2297, C = 0.2831, G = 0.2311, T = 0.2561; substitution rates AC = 1.2386, AG = 4.4108, AT = 0.9986, CG = 0.8412, CT = 7.1418, and GT = 1.0000. The gamma distribution shape parameter alpha was equal to 0.3965 and the Tree-Length was equal to 12.0800. Using BI analysis, the final average standard deviation of the split frequencies at the end of the total MCMC generations was estimated to be 0.00513. In terms of topology, the phylograms of the ML and BI analyses were similar (data not shown). The phylogram generated from the ML analysis is shown in Figure 4. Our phylogenetic tree was constructed concordantly and is supported by previous studies [4,18]. The phylogram separated all fungal strains in this study into four monophyletic clades with high BS and PP support values. These clearly formed distinct lineages from previous known *Exophiala* species with high BS and PP support values. The results of our study revealed that two fungal strains, namely SDBR-CMU417 and SDBR-CMU418 (introduced as *E. siamensis*), were clearly separated from the previously known species of *Exophiala*. Moreover, five fungal strains, SDBR-CMU404, SDBR-CMU405, SDBR-CMU406, SDBR-CMU407, and SDBR-CMU408 (introduced as *E. lamphunensis*), formed a sister taxon to the two strains SDBR-CMU415 and SDBR-CMU416 (described here as *E. saxicola*), with 80% and 1.00 BS and PP support values, respectively. Notably, *E. lamphunensis* and *E. saxicola* formed a sister clade to *E. xenobiotica*, with high BS (98%) and PP (1.0) support values. Moreover, our six strains, SDBR-CMU409, SDBR-CMU410, SDBR-CMU411, SDBR-CMU412, SDBR-CMU413, and SDBR-CMU414 (introduced as *E. lapidea*), formed a sister taxon to *E. moniliae* (BS = 99% and PP = 1.0). 

### 3.3. Taxonomic Descriptions 

**Figure 5:**
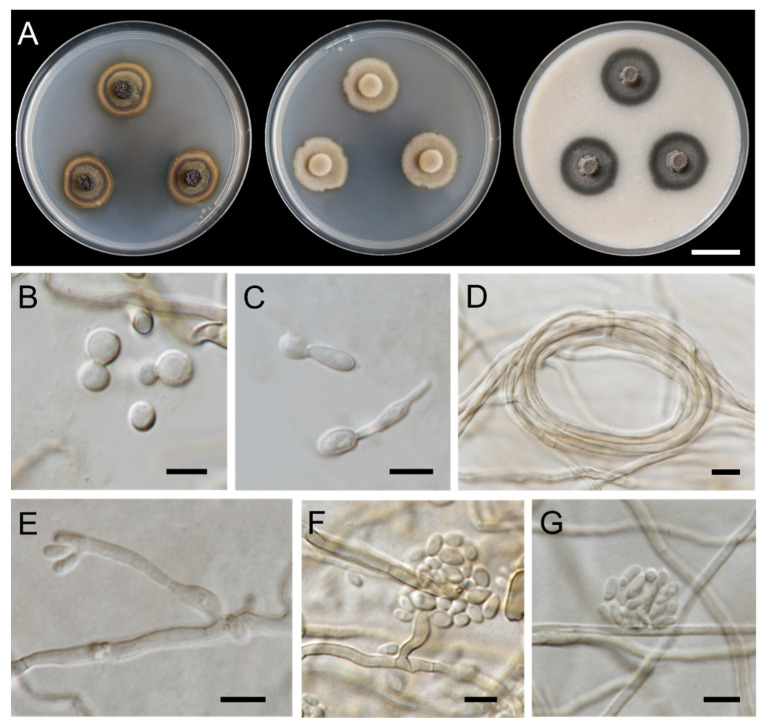
***Exophiala lamphunensis*** (SDBR-CMU404, holotype): (**A**) Colony at 25 °C for 28 days on PDA, MEA, and OA, respectively; (**B**) budding cells; (**C**) germinating cells; (**D**) hyphal coil; (**E**,**F**) subcylindrical conidiophore and conidiogenous cells; (**E**–**G**) conidia. Scale bars: (**A**) = 2 cm; (**B**–**G**) = 5 μm.

***Exophiala lamphunensis*** Thitla, J. Kumla and N. Suwannarach sp. nov. (Figure 5).

MycoBank No.: 844209.

Etymology: “*lamphunensis*”, referring to Lamphun Province, the original place of fungus isolation. 

Holotype: THAILAND, Lamphun Province, Mueang Lamphun District, Sribuaban Subdistrict, 18°32′11″ N 99°07′29″ E elevation 414 m, isolated from the rock of natural forest, July 2021, T. Thitla, dried culture: SDBR-LPN6_65; ex-type culture: SDBR-CMU404. 

GenBank: ON555798 (ITS), ON555813 (nrSSU), ON544242 (*tef*), ON544227 (*tub*), and ON544257 (*act*).

Culture characteristics: Colonies on PDA, MEA, and OA were described at 25 °C after 28 days of incubation (Figure 5A). Colonies on PDA reached 20–24 mm in diameter, restricted, circular, flat, velvety, and greyish green to dark green with greyish-green edges. Reverse dark green at the center and dull-green to greyish-green entire margin. Colonies on MEA attained a diameter of 23–26 mm, restricted, circular, raised, and velvety with dull-green aerial mycelium and entire margins. Reverse dark green to greyish green. Colonies on OA reached a diameter of 22–24 mm, restricted, circular, and velvety with greenish-grey and dark-green margins. Reverse dull green. A soluble dark-green pigment was observed around the fungal colonies on PDA. Budding cells initially abundant, hyaline, subspherical to ellipsoidal, 2.8–7.2 × 2.0–4.4 µm (mean = 4.6 × 3.3 µm, *n* = 50) (Figure 5B). Germinating cells abundant, hyaline, subspherical to ellipsoidal, 3.1–7.3 × 2.4–5.8 µm (mean = 5.0 × 3.5 µm, *n* = 50) (Figure 5C). Hyphae smooth-walled, pale olive-brown, 1.2–3.2 µm wide. Hyphal coils abundant while anastomoses absent (Figure 5D). Conidiophores short, subcylindrical, and intercalary of hyphae (Figure 5E). Conidiogenous cells erect, cylindrical with short annellated zones emerging from both the conidiophore and the terminal or the intercalary of the hyphae (Figure 5E,F). Conidia attached in tiny clusters, subhyaline, obovoidal, and 2.7–5.3 × 1.5–3.2 µm (mean = 3.8 × 2.1 µm, *n* = 50) (Figure 5E–G). Chlamydospores absent. Torulose hyphae up to 6 µm wide in appearance. Teleomorph were not found in any culture media.

Growth temperature: growth occurred within a range of 10–37 °C, optimum at 28 °C, while no growth at 4 and 40 °C.

Additional specimens examined: THAILAND, Lamphun Province, Mueang Lamphun District, Sribuaban Subdistrict, 18°32′11″ N 99°07′29″ E elevation 414 m, isolated from rock in dipterocarp forest, July 2021, isolated by T. Thitla: SDBR-CMU405, SDBR-CMU406, SDBR-CMU407, and SDBR-CMU408.

Known distribution: Lamphun Province, Thailand.

Note: Colonies on MEA of *E. lamphunensis* were similar to *E. nagquensis*, *E. oligosperma*, *E. saxicola*, and *E. xenobiotica*. However, *E. oligosperma*, *E. saxicola*, and *E. xenobiotica* differed from *E. lamphunensis* in the way they did not produce any soluble pigment on PDA [17,18,27]. The conidial sizes (4.8–10.4 × 2.6–5.0 µm) of *E. nagquensis* were larger than *E. lamphunensis* while *E. nagquensis* could grow at 4 *°C* [18]. The size of the budding cells and conidia of *E. lamphunensis* were within the range of *E. oligosperma*, *E. saxicola*, and *E. xenobiotica*. However, *E. oligosperma* and *E. saxicola* produced chlamydospores that differed from *E. lamphunensis*. Moreover, the optimum growth of *E. saxicola* was observed at 30 °C, which was higher than for *E. lamphunensis*. Notably, *E. xenobiotica* differed from *E. lamphunensis* in it has a slightly shorter conidial size (3.3–4.0 × 1.6–2.0 µm) and chlamydospore formation [17,27]. 

The phylogenetic analyses of the combined ITS, nrSSU, *tub*, *tef*, and *act* sequences confirmed that *E. lamphunensis* formed a monophyletic clade that clearly distinguished it from *E. nagquensis*, *E. oligosperma*, *E. saxicola*, and *E. xenobiotica*. *Exophiala lamphunensis* formed a sister clade to *E. saxicola*. However, sequence similarity and pairwise nucleotide comparison of tef data also showed that *E. lamphunensis* differs from *E. saxicola* in 97% and 3.1% (5/162 bp), respectively. Differences in the morphological characteristics and the optimum growing temperature were found between *E. lamphunensis* and *E. saxicola*. *Exophiala lamphunensis* produces soluble pigment on PDA and chlamydospore production is absent while this was not the case for *E. saxicola*. The slightly wider size of the germinating cells in *E. lamphunensis* (3.1–7.3 × 2.4.–5.8 µm) distinguished it from *E. saxicola* (3.6–6.0 × 1.9–3.7 µm). Additionally, *E. lamphunensis* had a lower optimum temperature (28 °C) than *E. saxicola* (30 °C). Therefore, *E. lamphunensis* and *E. saxicola* were considered as different species based on their morphological, optimal growth temperature, and tef sequence data.

**Figure 6:**
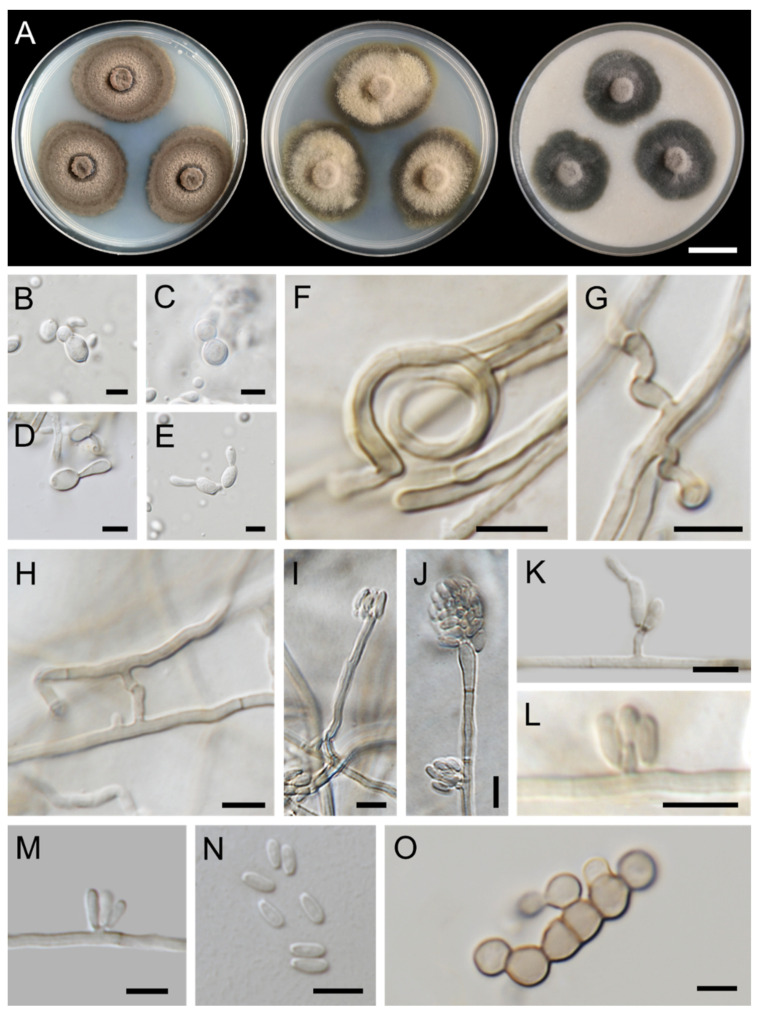
***Exophiala lapidea*** (SDBR-CMU409, holotype): (**A**) Colony at 25 °C for 28 days on PDA, MEA, and OA, respectively; (**B**,**C**) budding cells; (**D**,**E**) germinating cells; (**F**) hyphal coil; (**G**) spirally twisted hyphae; (**H**) anastomoses; (**I**) erect, cylindrical conidiophore; (**J**–**M**) conidial apparatus with conidia; (**I**–**N**) conidia; (**O**) torulose hyphae. Scale bars: (**A**) = 2 cm; (**B**–**O**) = 5 μm.

***Exophiala lapidea*** Thitla, J. Kumla and N. Suwannarach sp. nov. (Figure 6).

MycoBank No.: 844211.

Etymology: “*lapidea*” referring to the fungi being isolated from rock.

Holotype: THAILAND, Lamphun Province, Mueang Lamphun District, Sribuaban Subdistrict, 18°32′10″ N 99°07′30″ E elevation 407 m, isolated from the rock of natural forest, July 2021, T. Thitla, dried culture: SDBR-LPN8_9; ex-type culture: SDBR-CMU409.

GenBank: ON555803 (ITS), ON555818 (nrSSU), ON544247 (*tef*), ON544232 (*tub*), and ON544262 (*act*).

Culture characteristics: Colonies on PDA, MEA, and OA were described at 25 °C after 28 days of incubation (Figure 6A). Colonies on PDA grew to 35–48 mm in diameter, restricted, flat, velvety, greyish brown to dark brown, with black slime at the center. Reverse black with brown margin. Colonies on MEA reached 32–42 mm in diameter, restricted, flat, dull green to greyish green with aerial mycelium at the middle. Reverse dark green with deep-green margins. Colonies on OA grew to a diameter of 30–33 mm, restricted, circular, flat, velvety, greenish grey with aerial mycelium at the middle and dark-green edge. Reverse dark green. Budding cells abundant, hyaline, spherical or ellipsoidal, 2.8–5.1 × 2.2–4.7 µm (mean = 4.2 × 3.7 µm, *n* = 50) (Figure 6B,C). Germinating cells ellipsoidal, 4.6–8.4 × 2.5–5.3 µm (mean = 6.6 × 3.8 µm, *n* = 50) (Figure 6D,E). Hyphae smooth, thin-walled, pale olive-brown, usually spiral, 1.2–2.1 µm wide. Anastomoses and hyphal coil abundant (Figure 6F–H). Conidiophores pale olivaceous brown, erect, cylindrical, inserted laterally on hyphae (Figure 6I). Conidiogenous cells erect, cylindrical, with short annellated zones emerging from hyphae, terminal or intercalary (Figure 6J–L). Conidia hyaline, thin-walled, obovoidal, 2.9–7.0 × 0.9–2.4 µm (mean = 4.3 × 1.5 µm, *n* = 50) with inconspicuous basal scars (Figure 6I–N). Chlamydospores absent. Torulose hyphae appeared, up to 7 μm wide (Figure 6O). Teleomorph not found in any culture media.

Growth temperature: growth occurred within a range of 10–37 °C, optimum at 28 °C, while no growth at 4 and 40 °C.

Additional specimens examined: THAILAND, Lamphun Province, Mueang Lamphun District, Sribuaban Subdistrict, 18°32′10″ N 99°07′30″ E elevation 407 m, isolated from rock in dipterocarp forest, July 2021, isolated by T. Thitla: SDBR-CMU410, SDBR-CMU411, SDBR-CMU412, SDBR-CMU413, and SDBR-CMU414.

Known distribution: Lamphun Province, Thailand.

Note: The colony characteristics of *E. lapidea* were similar to those of *E. aquamarine*, *E. cancerae*, and *E. eucatypticola*. However, the conidial size of *E. lapidea* (2.9–7.0 × 0.9–2.4 µm) was clearly smaller than *E. aquamarine* (6.7–19.2 × 4.0–4.8 µm) [3]. The wider size of the conidia in *E. cancerae* (4.9–8.0 × 2.7–4.8) and *E. eucalypticola* (4.0–7.0 × 2.0–3.0 µm) clearly distinguished them from *E. lapidea* [3,22]. Moreover, *E. cancerae* and *E. eucalypticola* could effectively grow at 4 °C.

The multi-gene phylogenetic analyses (ITS, nrSSU, *tub*, *tef*, and *act* genes) confirmed that *E. lapidea* formed a monophyletic clade that clearly separated it from the other previous known *Exophiala* species and closely related species. A phylogram showed that *E. lapidea* formed a sister taxon to *E. moniliae* (Figure 4). However, the shorter size of conidia in *E. moniliae* (2.3–3.9 × 1.6–2.2 µm) clearly distinguished it from *E. lapidea* [15].

**Figure 7:**
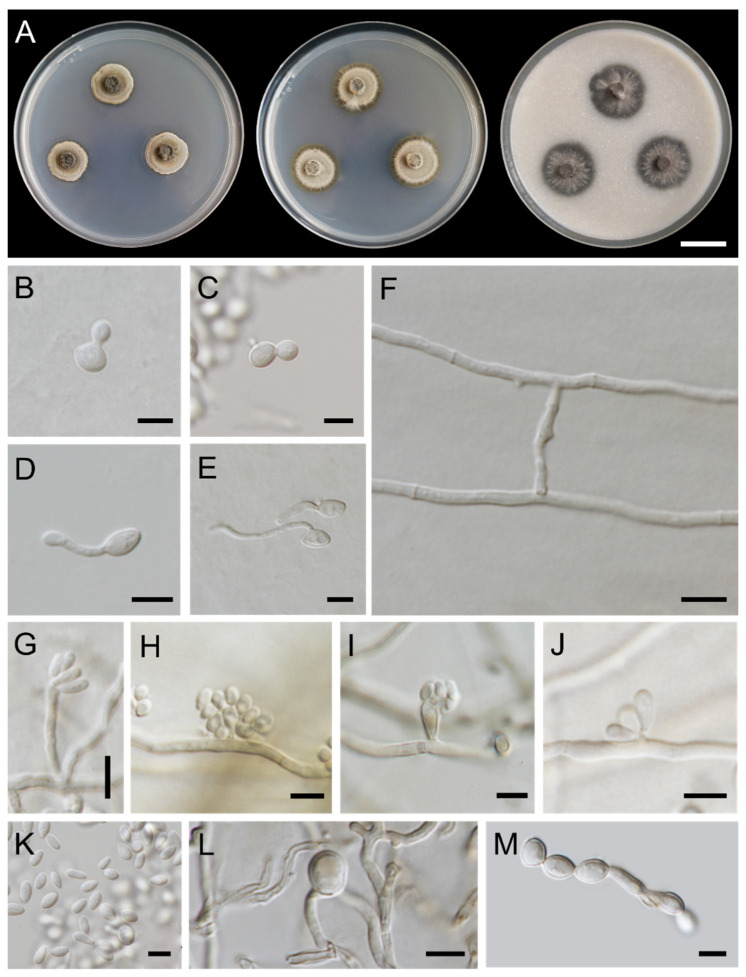
***Exophiala saxicola*** (SDBR-CMU415, holotype): (**A**) colony at 25 °C for 28 days on PDA, MEA, and OA, respectively; (**B**,**C**) budding cells; (**D**,**E**) germinating cells; (**F**) anastomoses; (**G**) erect, cylindrical conidiophore; (**H**,**I**) obovoidal conidiogenous cells with obovoidal conidia; (**J**) conidial apparatus with conidia; (**G**–**K**) conidia; (**L**) chlamydospore; (**M**) torulose hyphae. Scale bars: (**A**) = 2 cm; (**B**–**M**) = 5 μm.

***Exophiala saxicola*** Thitla, N. Suwannarach and S. Lumyong sp. nov. (Figure 7).

MycoBank No.: 844212.

Etymology: “*saxicola*” referring to a stone inhabitant. 

Holotype: THAILAND, Lamphun Province, Mueang Lamphun District, Sribuaban Subdistrict, 18°32′11″ N 99°07′30″ E elevation 413 m, isolated from the rock of natural forest, July 2021, T. Thitla, dried culture: SDBR-LPN6_71; ex-type culture: SDBR-CMU415.

GenBank: ON555809 (ITS), ON555824 (nrSSU), ON544253 (*tef*), ON544238 (*tub*), and ON544268 (*act*)

Culture characteristics: Colonies on PDA, MEA, and OA were described at 25 °C after 28 days of incubation (Figure 7A). All culture media restricted, circular, flat, and velvety. On PDA, PDA grew to 16–18 mm in diameter, dull green and dark green in reverse. Colonies on MEA reached 20–22 mm in diameter, dull-green and greyish-green margins. Reverse greyish green to dark green. Colonies on OA attained a diameter of 22–24 mm, greenish grey to dark green. Reverse dark green. Budding cells initially abundant, hyaline, subspherical to ellipsoidal, 4.0–7.0 × 2.7–5.2 µm (mean = 5.6 × 3.8 µm, *n* = 50) (Figure 7B,C). Germinating cells abundant, hyaline, ellipsoidal, 3.6–6.0 × 1.9–3.7 µm (mean = 4.8 × 2.6 µm, *n* = 50) (Figure 7D,E). Hyphae smooth-walled, pale olive-brown, 1.1–3.3 µm wide. Anastomoses abundant (Figure 7F). Conidiophores pale olivaceous brown, erect, cylindrical (Figure 7G). Conidiogenous cells obovoidal to clavate with short annellated zones, intercalary or terminal of hyphae (Figure 7H,I). Conidia adhering in small groups, hyaline, obovoidal, 2.8–6.2 × 1.3–3.5 µm (mean = 4.4 × 2.3 µm, *n* = 50) (Figure 7G–K). Chlamydospores are presented, subspherical, brown, 4.1–8.1 × 3.3–7.2 µm (Figure 7L). Torulose hyphae appeared up to 5 μm in width (Figure 7M). Teleomorph not found in any culture media. 

Growth temperatures: growth occurred within a range of 10–37 °C, optimum at 30 °C, while no growth at 4 and 40 °C.

Additional specimens examined: THAILAND, Lamphun Province, Mueang Lamphun District, Sribuaban Subdistrict, 18°32′11″ N 99°07′30″ E elevation 413 m, isolated from rock in dipterocarp forest, July 2021, isolated by T. Thitla: SDBR-CMU416.

Known distribution: Lamphun Province, Thailand.

Note: The colony characteristics of *E. saxicola* on MEA were similar to those observed for *E. xenobiotica*, *E. nagquensis*, *E. oligosperma*, and *E. lamphunensis*. The production of soluble pigment on PDA was observed as was an absence of chlamydospore formation in *E. lamphunensis*, which clearly distinguished it from *E. saxicola*. The budding cells of *E. saxicola* (4.0–7.0 × 2.7–5.2 µm) were larger than the budding cells of *E. oligosperma* (3.0 × 2.5 µm) [17]. Notably, the small size of the germinating cells in *E. saxicola* (3.6–6.0 × 1.9–3.7 µm) clearly distinguished it from *E. xenobiotica* (7.0–10.0 × 3.0–5.0 µm) [27] and *E. oligosperma* (6.0 × 5.0 µm) [17]. Moreover, the conidia size of *E. nagquensis* (4.8–10.4 × 2.6–5.0 µm) was larger than *E. saxicola* [18]. The optimal growth temperature of *E. saxicola* (30 °C) distinguished it from *E. lamphunensis* (28 °C). Moreover, the maximum growth temperature of *E. saxicola* (37 °C) was higher than for *E. xenobiotica* (33–36 °C) [27] and *E. nagquensis* (28 °C) [18].

The phylogenetic analyses of the combined ITS, nrSSU, *tub*, *tef*, and *act* sequences confirmed that *E. saxicola* formed a monophyletic clade that clearly distinguished it from the other closely related species, namely *E. nagquensis*, *E. oligosperma*, and *E. xenobiotica*. Furthermore, *E. saxicola* formed a sister clade to *E. lamphunensis*. However, differences in the morphological characteristics, optimal growth temperature, and tef sequence data of *E. saxicola* and *E. lamphunensis* were observed and described above.

**Figure 8:**
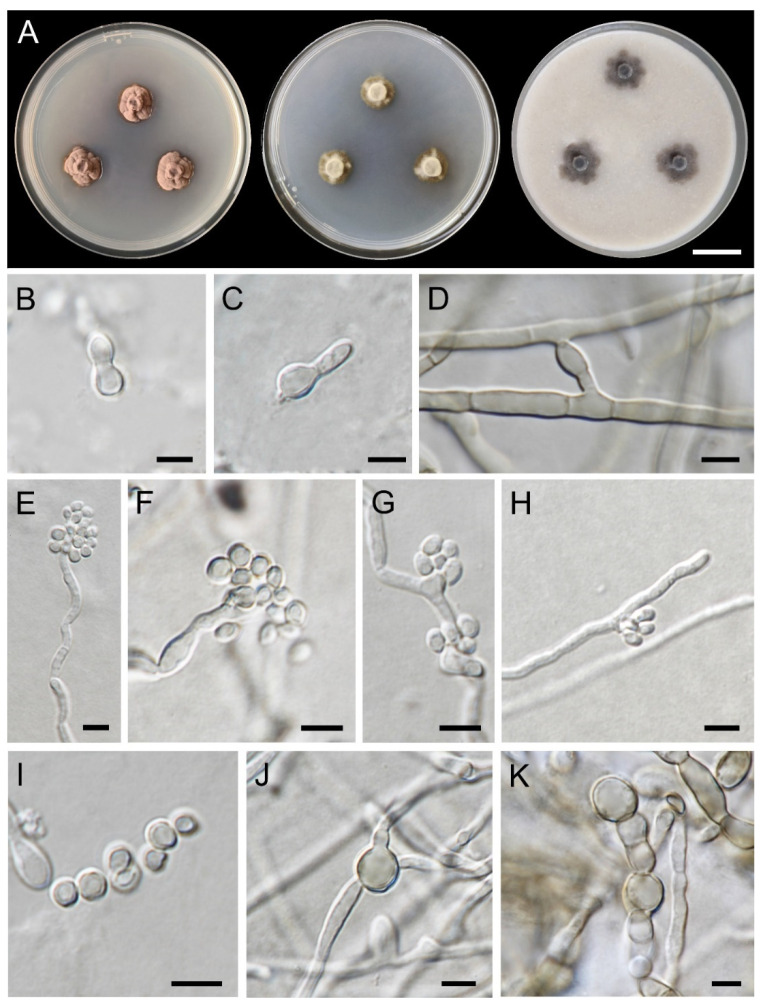
***Exophiala siamensis*** (SDBR-CMU417, holotype): (**A**) colony at 25 °C for 4 weeks on PDA, MEA, and OA respectively; (**B**) budding cells; (**C**) germinating cells; (**D**) anastomoses; (**E**–**I**) conidial apparatus with subspherical conidia; (**J**) chlamydospore; (**K**) torulose hyphae. Scale bars: (**A**) = 2 cm; (**B**–**G**) = 5 μm.

***Exophiala siamensis*** Thitla, J. Kumla and N. Suwannarach sp. nov. (Figure 8).

MycoBank: 844213.

Etymology: “*siamensis*” referring to Siam (old name of Thailand), where this fungus was found.

Holotype: THAILAND, Sukhothai Province, Si Satchanalai District, 17°32′58″ N 99°29′49″ E elevation 153 m, isolated from the rock of natural forest, June 2021, T. Thitla, dried culture: SDBR-SKT3_3; ex-type culture: SDBR-CMU417. 

GenBank: ON555811 (ITS), ON555826 (nrSSU), ON544255 (*tef*), ON544240 (*tub*), and ON544270 (*act*).

Culture characteristics: Colonies on PDA, MEA, and OA were described at 25 °C after 28 days of incubation (Figure 8A). Colonies on PDA were 14–21 mm in diameter, restricted, irregular, convex in elevation, and velvety with brownish-grey and dark-brown margins. Reverse black. Colonies on MEA and OA restricted, circular, flat, velvety. Colonies on MEA grew to 9–11 mm in diameter with dark-green to greyish-green and white margins. Reverse dark green. Colonies on OA reached a diameter of 15–16 mm with dark-green and greyish-green margins. Reverse black and olive margin. Budding cells rarely, hyaline, subspherical, 5.8–7.6 × 4.3–5.9 µm (mean = 6.7 × 5.3 µm, *n* = 50) (Figure 8B). Germinating cells ovoidal or obovoidal, 4.7–6.2 × 3.2–4.8 µm (mean = 5.5 × 3.9 µm, *n* = 50) (Figure 8C). Hyphae smooth, thin-walled, pale olive-brown, 1.2–3.0 µm in width, producing conidia apically and laterally. Anastomoses presence (Figure 8D). Conidiophores short, erect, cylindrical (Figure 8E). Conidiogenous cells cylindrical to ellipsoidal, terminal or intercalary (Figure 8F–H). Conidia hyaline, thin-walled, subspherical, 1.9–3.5 × 1.5–3.2 µm (mean = 2.7 × 2.2 µm, *n* = 50) (Figure 8E–I). Chlamydospores subspherical, pale brown, 7.4–16.5 × 3.1–6.7 µm (Figure 8J). Torulose hyphae appeared up to 6 μm in width (Figure 8K). Teleomorph not found in any culture media. 

Growth temperatures: growth occurred within a range of 10–30 °C, optimum at 20 °C, while no growth at 4, 35, 37, and 40 °C.

Additional specimens examined: THAILAND, Sukhothai Province, Si Satchanalai District, 17°32′58″ N 99°29′49″ E elevation 153 m, isolated from rock in dipterocarp forest, June 2021, isolated by T. Thitla: SDBR-CMU418.

Known distribution: Sukhothai Province, Thailand.

Note: Morphologically, the colony characteristics of *E. siamensis* were similar to *E. ellipsoidea*, *E. brunnea*, *E. polymorpha*, and *E. radicis*. However, the wider size of the budding cells in *E. siamensis* (5.8–7.6 × 4.3–5.9 µm) clearly separated it from *E. polymorpha* (4.0–6.0 × 2.5–4.0 µm) [8]. The conidial size of *E. siamensis* (1.9–3.5 × 1.5–3.2 µm) was smaller than *E. radicis* (4.0–11.0 × 2.0–5.0 µm) [5]. In addition, the conidia size of *E. siamensis* was clearly shorter than *E. brunnea* (4.5–10.0 × 2.0–3.0 µm) [3], *E. ellipsoidea* (2.1–6.4 × 1.1–1.0 µm) [18], and *E. polymorpha* (3.5–4.0 × 1.5–2.5 µm) [8]. *Exophiala siamensis* produced chlamydospores that were different from *E. polymorpha* and *E. radices* [5,8]. The maximum growth temperature of *E. ellipsoidea* (33 *°C*) and E. polymorpha (30 °C) was higher than for *E. siamensis* (30 °C) [8,18]. The minimum growth temperature of *E. brunnea* (4–9 °C) was lower than *E. siamensis* (10 °C) [3].

Moreover, a multi-gene phylogenetic analysis confirmed that *E. siamensis* formed a well-supported monophyletic clade that was distinctly separated from other *Exophiala* species.

## 4. Discussion

Species of the genus *Exophiala* are known to be widely distributed around the world [3,7,33,151]. The traditional identification of *Exophiala* species has primarily been based on morphological characteristics [1,15,152]. However, identification can be difficult because some of the polymorphic characteristics are shared and some species have a similar appearance [3,5,8,15,89]. As a result, some previously identified *Exophiala* species were then transferred from the genera *Graphium*, *Haplographium*, *Hormiscium*, *Phaeococcomyces*, *Phaeococcus*, *Phialophora*, *Pullularia*, *Sarcinomyces*, *Sporocybe*, *Trichosporum*, and *Torula* [3,11,12,13,14,15,16,17]. Therefore, a combination of morphological and multi-gene data was used to concretely identify the *Exophiala* species [3,4,5,9,18]. Prior to conducting our study, a total of 63 species had been validated, published, and accepted into the genus *Exophiala*. 

In this study, four new species of *Exophiala*, consisting of *E. lamphunensis*, *E. lapidea***,** *E. saxicola*, and *E. siamensis*, were introduced. The different morphological characteristics identified between the four new species indicate that only *E*. *lamphunensis* produced soluble pigments around the colonies on PDA. Chlamydospore formations were observed in *E. saxicola* and *E. siamensis*, but this was not the case for *E. lamphunensis* and *E. lapidea*. Additionally, the budding cells of *E. siamensis* were larger and wider than those of *E. lapidea* and *E. lamphunensis*. However, the germinating cells and conidia of our four species were not observed to be different. The optimum growth temperature of *E. lapidea* and *E. saxicola* was 30 °C, which was higher than for *E*. *lamphunensis* (28 °C) and *E*. *siamensis* (28 °C). Additionally, the maximum growth temperature of *E. siamensis* (30 °C) was lower than for the other three new species (37 °C). Subsequently, our phylogenetic analyses of the combined five genes (ITS, nrSSU, *tub*, *tef*, and *act*) revealed that the four new species formed distinct lineages within the genus *Exophiala*. Therefore, a combination of the morphological characteristics and the molecular analyses conducted in our study strongly support the recognition of four new *Exophiala* species.

*Exophiala* species have been isolated in various habitats throughout the world as shown in Table 1. Several *Exophiala* species have been identified as potential agents of human and animal diseases. However, in some studies, certain *Exophiala* species have been employed in agricultural and biotechnological applications. In this study, four new *Exophiala* species were isolated from rock samples collected from natural forests located in northern Thailand. Our findings are similar to those of previous studies, which reported that some *Exophiala* species (e.g., *E*. *bonaiae*, *E*. *cinerea*, *E*. *clavispora*, *E*. *ellipsoidea*, and *E*. *nagquensis*) have been successfully isolated from rock samples. However, there have been no prior reports involving investigations of rock-inhabiting fungi in Thailand. Therefore, our study is the first of its kind to report on the discovery of *Exophiala* on rocks in Thailand. Prior to our study, a total of three *Exophiala* species (*E*. *dermatitidis*, *E*. *jeanselmei*, and *E*. *spinifera*) were known from Thailand [43,46,67]. Therefore, the successful identification of the *Exophiala* species in this study has increased the number of species found in Thailand to 7 species and has led to 67 global species. The outcomes of this present study will provide scientists and researchers with valuable information that can stimulate deeper investigations of rock-inhabiting fungi in Thailand. Ultimately, these findings will help researchers gain a better understanding of the distribution and ecology of *Exophiala*.

## Figures and Tables

**Table 1:** Global distribution and isolation resources of *Exophiala* species.

Species	Isolation Resources	Location	Reference
*Exophiala abietophila*	Silver fir (*Abies alba*)	Norway	[21]
*Exophiala alcalophila*	Soil, soap container, washing machine, bathwater from households, and human skin	Brazil, Denmark, Germany, Japan, and Ukraine	[3,75,76]
*Exophiala angulospora*	Polluted soil, drinking water, Tilia wood, fish nursery, weedy seadragon, lumpfish skin and spleen, olive flounder (*Paralichthys olivaceus*), Atlantic cod (*Gadus morhua*), and human skin	Brazil, Denmark, Germany, Ireland, Japan, Netherlands, Norway, Russia, Scotland, and the USA	[3,77,78,79,80,81]
*Exophiala aquamarina*	Clown fish, leafy sea dragon, little tunnyfish, lumpfish, sand lance, weedy seadragon, and winter flounder	Canada, the UK, and the USA	[3,7]
*Exophiala arunalokei*	Subcutaneous lesion on human	India	[20]
*Exophiala asiatica*	Tonsil tissue of human	China	[30,32]
*Exophiala attenuata*	Soil, nasal granuloma of cat, cutaneous phaeohyphomycosis of cat, and human disease	Colombia, France, Germany, and the USA	[33,82,83,84]
*Exophiala bergeri*	Eye and skin of human	Brazil, Canada, Japan, Hong Kong, the UK, and the USA	[6,7,31,85]
*Exophiala bonariae*	Marble	Italy	[69]
*Exophiala brunnea*	Leaf of *Acacia karroo*	South Africa	[3]
*Exophiala calicioides*	Rotten wood	Japan	[68]
*Exophiala campbellii*	Subcutaneous lesion (foot ganglion) of human and human chest nodule	Germany and the UK	[7,70]
*Exophiala cancerae*	Water, water from tank, fruit drink, dialysis water Mangrove crab (*Ucides cordatus*), liver of green toad, sputum of human, and human finger	Australia, Brazil, Canada, Germany, Hong Kong, Israel, Netherlands, the UK, and the USA	[3,7,31,86,87,88]
*Exophiala capensis*	Leaf of *Phaenocoma prolifera*	Canada and South Africa	[7,30,31,32,33,68,69,70,82,83,84,85,86,87,88,89]
*Exophiala castellanii*	Decaying timber joinery, spoilt apple juice, drinking water, ice water, nematode, and human skin	Denmark, Germany, Netherlands, Sri Lanka, Switzerland, and the UK	[3,27]
*Exophiala cinerea*	Rock	China	[18]
*Exophiala clavispora*	Rock	China	[18]
*Exophiala crusticola*	Biological soil crust	the USA	[90]
*Exophiala dermatitidis*	Soil, dishwasher’s rubber, wood, internal organs of bat, chromoblastomycosis, knee fluid, lung, finger, and central nervous system fluid of human	Angola, Brazil, China, Finland, Germany, Hong Kong, Iran, Iraq, Japan, Korea, Malaysia, Mauritius, Qatar, Slovenia, South Africa, Taiwan, Thailand, Turkey, the UK, the USA, and Venezuela	[7,15,16,31,32,45,46,47,48,49,50,51,52,53]
*Exophiala dopicola*	Loblolly pine (*Pinus taeda*)	the USA	[91]
*Exophiala ellipsoidea*	Rock	China	[18]
*Exophiala embothrii*	Rhizosphere of *Embothrium coccineum*	Chile	[92]
*Exophiala encephalarti*	On leaves of *Encephalartos transvenosus*	South Africa	[93]
*Exophiala equina*	Soil, drinking water, bottled water, water from water machine, water system of packaging machine, wastewater, dialysis water bathroom-flask, bathroom-plate, silica gel, root mycorrhiza, Tilia root, *Populus tremuloides*, *Cephalanthera damasonium*, *Phragmitis australis*, *Olea* twig, nematode cyst (*Heterodera* sp.), subcutaneous infection of horse, Galapagos turtle, human stool, human sputum, human eye, and skin of human	Australia, Brazil, Canada, Denmark, Germany, Italy, Japan, Netherlands, Korea, the UK, and the USA	[3,7,10,87,94,95,96,97,98]
*Exophiala eucalypti*	Leaves of *Eucalyptus* sp.	South Africa	[99]
*Exophiala eucalypticola*	Leaf of *Eucalyptus obliqua*	Australia	[22]
*Exophiala eucalyptorum*	Leaf of *Eucalyptus* sp.	New Zealand	[23]
*Exophiala exophialae*	Soil, straw in armadillo’s burrow (*Dasypus septemcinctus*)	Colombia and Uruguay	[6,15,34]
*Exophiala frigidotolerans*	Soil	Ecuador	[100]
*Exophiala halophila*	Salty water, human skin, and human nail	Germany and the USA	[3]
*Exophiala heteromorpha*	Wood and human	Sweden and the USA	[7,15]
*Exophiala hongkongensis*	Big toenail infection of human	China and Hong Kong	[31]
*Exophiala italica*	*Cytisus scoparius* on dead branch	Italy	[24]
*Exophiala jeanselmei*	Subcutaneous abscesses, skin lesion, eumycetoma of human, peritoneal dialysis fluid, human blood, human sputum, and human eye	Australia, Bangladesh, Brazil, Canada, China, Costa Rica, France, Hong Kong, Jamaica, Japan, Martinique, Pakistan, Paraguay, Peru, Philippines, Saudi Arabia, Thailand, Trinidad, the UK, Uruguay, and the USA	[6,17,27,31,32,33,34,54,55,56,57,58,59,60,61,62,63,64,65,66,67]
*Exophiala lacus*	Lake water and river sediments	Netherlands and Spain	[3,71]
*Exophiala lavatrina*	Domestic bathroom	the UK	[7]
*Exophiala lecanii-corni*	*Lecanium corni*, domestic bathroom, dialysis fluid, subcutaneous abscess, knee cyst, skin lesion, eye sclera, finger fluid, skin scales, and human nail	Austria, Germany, Hong Kong, Japan, Netherlands, the UK, and the USA	[27,31,57,101,102]
*Exophiala lignicola*	*Quercus* sp.	Ukraine	[21]
*Exophiala macquariensis*	Island soil	Australia	[2]
*Exophiala mali*	Inner fruit tissue of *Malus* sp.	South Africa	[92]
*Exophiala mansonii*	*Populus tremula*	Sweden	[15]
*Exophiala mesophila*	Shower joint, swimming pool, dental waterline, bathroom, contact lens, phaeohyphomycotic cyst, subcutaneous nodule biopsy, immunosuppressed, bronchial endoscopy, finger, sinus, hip joint, hair, and nasal tissue of human	Brazil, France, Germany, Netherlands, the UK, and the USA	[3,7,85]
*Exophiala moniliae*	Branch of *Quercus* sp., sludge in bathroom drainpipes, and medicated bathwater	Australia, Japan, and Russia	[15,76,103]
*Exophiala nagquensis*	Rock	China and Tibet	[18]
*Exophiala nidicola*	Nest of bird	Spain	[72]
*Exophiala nishimurae*	Bark and human skin	the USA and Venezuela	[17,33]
*Exophiala oligosperma*	Soil, wood, swimming pool, water, polluted water, river sediments, sauna, silicone solution, ear swab, plastic foil, prosthetic contact lenses, cerebral mycosis, subcutaneous abscess, thigh abscess, skin lesion, sphenoid tumor, lung, sinus, and human sputum	Austria, Brazil, Canada, Finland, France, Germany, Hong Kong, Italy, Japan, Netherlands, Spain, Switzerland, the UK, Ukraine, the USA, and Venezuela	[6,7,17,31,34,57,71,104,105,106]
*Exophiala opportunistica*	Drinking water, rhizosphere (*Triticum aestivum*), polyvinyl alcohol, human nail, and human foot	Australia, Denmark, Germany, and Netherlands	[3]
*Exophiala palmae*	Decaying shell of babassu coconut (*Orbignya phalerata*)	Brazil	[107]
*Exophiala phaeomuriformis*	Natural hot spring, sauna, tile floor of swimming pool, bathroom tap, bathroom sink, cutaneous mycosis, blood culture, external ear channel, oral mucosa, nail, and human sputum,	Austria, Canada, Czech Republic, Germany, Japan, Netherlands, Slovenia, the UK, and the USA	[7,16,108]
*Exophiala pisciphila*	Swimming pool, water pipe, dialysis water, catfish (*Ictalurus punctatus*), Potbelly seahorse, crocodile, and human	Brazil, Germany, Japan, Israel, and the USA	[3,7,87,109]
*Exophiala polymorpha*	Subcutaneous lesion of human	the USA	[8]
*Exophiala prostantherae*	Leaves of *Prostanthera* sp.	Australia	[92]
*Exophiala pseudooligosperma*	Karst rocky desertification mountain soil	China	[4]
*Exophiala psychrophila*	Atlantic salmon smolt (*Salmo salar*)	Ireland and Norway	[3]
*Exophiala quercina*	Dead wood of *Quercus* sp.	Germany	[73]
*Exophiala radicis*	Soil, root endophyte of *Microthlaspi perfoliatum*, plant roots, *Olea* sp. twig, nematode cyst (*Heterodera* sp.), toenail, tinea on leg, and foot of human	Bulgaria, Denmark, France, Germany, Italy, the Netherlands, and Spain	[5,71]
*Exophiala salmonis*	Drinking water, drinking water tap and cerebral mycetoma of fingerling trout (*Salmo clarkii*)	Canada and the Netherlands	[1]
*Exophiala sideris*	Oak railway tie, creosoted tie, gold mine, and surface of wild berries of *Sorbus aucuparia*	the Netherlands and Poland	[9]
*Exophiala spartinae*	*Spartina alterniflora* root tissue in saltwater marsh	the USA	[110]
*Exophiala spinifera*	Soil, palm tree, wood, nest of *Anumbius annumbi*, armadillo burrow, maize kernel, apple juice, rotten cactus, skin lesion, foot abscess, neck lymph node, human sputum, and bark nasal granuloma of human	Antarctic, Argentina, Australia, Brazil, China, Colombia, Egypt, Germany, India, Mexico, Papua New Guinea, Senegal, Thailand, the UK, Uruguay, the USA, and Venezuela	[6,32,33,34,35,36,37,38,39,40,41,42,43,44]
*Exophiala tremulae*	*Populus tremuloides* roots	Canada	[111]
*Exophiala xenobiotica*	Soil, wood, oil sludge, chromoblastomycosis on back, phaeomycotic cyst, subcutaneous cyst, elbow pus, and skin lesions	Antarctic, Australia, Brazil, Canada, Germany, Hong Kong, Japan, the Netherlands, New Zealand, Switzerland, Sweden, the UK, the USA, and Venezuela	[6,7,27,31,34,57,74]

**Table 2:** List of the primers, primer sequences, and annealing temperatures used for PCR amplification in each target gene.

Target Gene	Primer	Primer Sequence (5′–3′)	Annealing Temperature (°C)	Reference
*act*	Act1	TGGGACGATATGGAIAAIATCTGGCA	52	[124]
	Act5ra	TTAGAAGCACTTNCGGTG	52	[124]
ITS	ITS4	TCCTCCGCTTATTGATATGC	55	[125]
	ITS5	GGAAGTAAAAGTCGTAACAAGG	55	[125]
nrSSU	NS1	GTAGTCATATGCTTGTCTC	55	[125]
	NS4	CTTCCGTCAATTCCTTTAAG	55	[125]
*tef*	EF1-728F	CATCGAGAAGTTCGAGAAGG	57	[126]
	EF1-986R	TACTTGAAGGAACCCTTACC	57	[126]
*tub*	Bt2a	GGTAACCAAATCGGTGCTGCTTTC	52	[127]
	Bt2b	ACCCTCAGTGTAGTGACCCTTGGC	52	[127]

**Table 3:** DNA sequences used in the molecular phylogenetic analysis.

Species	Strains	GenBank Accession No.					References
		ITS	nrSSU	*tub*	*tef*	*act*	
*Exophiala abietophila*	CBS 145038 ^T^	NR163357	–	–	–	–	[21]
	CBS 520.82 ^T^	JF747041	JN856010	JN112423	JN128771	JN112379	[3]
	CBS 122256	JF747044	–	JN112425	JN128773	JN112381	[3]
*Exophiala angulospora*	CBS 482.92 ^T^	JF747046	JN856011	JN112426	JN128780	JN112383	[3]
	CBS 120272	JF747045	–	JN112427	JN128781	JN112382	[3]
*Exophiala aquamarina*	CBS 119918 ^T^	JF747054	JN856012	JN112434	–	JN112388	[3]
	CBS 119916	JF747055	–	JN112435	–	JN112389	[3]
*Exophiala arunalokei*	NCCPF106033	MW724320	–	–	–	–	[20]
*Exophiala asiatica*	CBS 122847 ^T^	NR111332	–	–	–	–	[30]
	CBS 122848	MW222182	–	–	–	–	[30]
*Exophiala attenuata*	CBS 101540 ^T^	AF549446	–	–	–	–	[33]
	UTHSC87-80	EF025392	–	–	–	–	[130]
*Exophiala bergeri*	CBS 353.52 ^T^	EF551462	FJ358308	EF551497	EF551524	EF551464	[131]
*Exophiala bonariae*	CBS 139957 ^T^	JX681046	–	–	–	–	[69]
*Exophiala brunnea*	CBS 587.66 ^T^	JF747062	JN856013	JN112442	JN128783	JN112393	[3]
*Exophiala calicioides*	JCM6030	–	AB007655	–	–	–	[132]
*Exophiala campbellii*	NCPF 2274	LT594703	–	–	LT594739	–	[7]
*Exophiala cancerae*	CBS 120420 ^T^	JF747064	–	JN112444	JN128800	JN112394	[3]
	CBS 117491	KF928439	–	KF928567	JN128799	JN112396	[3]
*Exophiala capensis*	CBS 128771 ^T^	JF499841	–	–	–	–	[89]
*Exophiala castellanii*	CBS 158.58 ^T^	JF747070	JN856014	KF928586	JN128766	–	[3,133]
	CBS 120913	JF747144	–	JN112506	JN128750	–	[3]
*Exophiala cinerea*	CGMCC 3.18778 ^T^	MG012695	MG012724	MG012745	MG012704	MG012714	[18]
	CGMCC 3.18779	MG012696	MG012725	MG012746	MG012705	MG012715	[18]
*Exophiala clavispora*	CGMCC 3.17512	KP347940	MG012733	KP347931	KP347909	MG012712	[18]
	CGMCC 3.17517 ^T^	KP347942	KP347967	KP347932	KP347911	KP347893	[18]
*Exophiala crusticola*	CBS 119970 ^T^	AM048755	KF155199	–	–	–	[90,134]
	HM136	MK281393	–	–	–	–	Unpublished
*Exophiala dermatitidis*	CBS 207.35 ^T^	AF050269	–	KF928572	–	–	[133,134,135]
	CBS 120473	MF320159	–	MF320217	MF320196	–	[43]
*Exophiala dopicola*	CBS 537.94 ^T^	MH862483	–	–	–	–	[136]
*Exophiala ellipsoidea*	CGMCC 3.17348 ^T^	KP347955	KP347965	KP347921	KP347901	MG012713	[18]
	CGMCC 3.17522	KP347954	MG012735	KP347919	–	KP347884	[18]
*Exophiala embothrii*	CBS 146558 ^T^	NR171982	–	MW055976	MW055980	–	[92]
*Exophiala encephalarti*	CBS 128210 ^T^	HQ599588	–	–	–	–	[93]
*Exophiala equina*	CBS 119.23 ^T^	JF747094	JN856017	JN112462	JN128814	JN112401	[3]
	CBS 120906	JF747093	–	JN112461	JN128813	JN112400	[3]
*Exophiala eucalypti*	CBS 142069	KY173411	–	–	–	–	[99]
*Exophiala eucalypticola*	CBS 143412 ^T^	NR158438	–	MH108039	MH108016	–	[22]
*Exophiala eucalyptorum*	CBS 121638 ^T^	NR132882	KC455302	KC455228	–	–	[137]
	CPC 11261	EU035417	–	–	–	–	[23]
*Exophiala exophialae*	CBS 668.76 ^T^	AY156973	KX822287	EF551499	EF551526	EF551466	[33,138]
	CBS 671.76	AY156975	–	EF551500	EF551525	EF551467	[33]
*Exophiala frigidotolerans*	CBS 146539 ^T^	LR699566	–	–	–	–	[100]
*Exophiala halophila*	CBS 121512 ^T^	NR111628	NG062077	JN112473	JN128774	–	[3]
*Exophiala heteromorpha*	CBS 232.33 ^T^	AY857524	–	–	–	–	[139]
	U THSC87-67	EF025400	–	–	–	–	[130]
*Exophiala hongkongensis*	CBS 131511	JN625231	–	JN625236	JN625246	JN625241	[31]
*Exophiala italica*	MFLUCC16-0245 ^T^	KY496744	KY501114	–	KY514393	-	[24]
*Exophiala jeanselmei*	CBS 507.90 ^T^	AY156963	FJ358310	EF551501	EF551530	-	[33,131]
	CBS 528.76	AY857530	–	EF551502	EF551531	EF551469	[139]
*Exophiala lacus*	FMR 3995	KU705830	–	–	–	–	[71]
	CBS 117497 ^T^	JF747110	–	–	JN128776	JN112407	[3]
*Exophiala lamphunensis*	**SDBR-CMU404 ^T^**	**ON555798**	**ON555813**	**ON544227**	**ON544242**	**ON544257**	**This study**
	**SDBR-CMU405**	**ON555799**	**ON555814**	**ON544228**	**ON544243**	**ON544258**	**This study**
	**SDBR-CMU406**	**ON555800**	**ON555815**	**ON544229**	**ON544244**	**ON544259**	**This study**
	**SDBR-CMU407**	**ON555801**	**ON555816**	**ON544230**	**ON544245**	**ON544260**	**This study**
	**SDBR-CMU408**	**ON555802**	**ON555817**	**ON544231**	**ON544246**	**ON544261**	**This study**
*Exophiala lapidea*	**SDBR-CMU409 ^T^**	**ON555803**	**ON555818**	**ON544232**	**ON544247**	**ON544262**	**This study**
	**SDBR-CMU410**	**ON555804**	**ON555819**	**ON544233**	**ON544248**	**ON544263**	**This study**
	**SDBR-CMU411**	**ON555805**	**ON555820**	**ON544234**	**ON544249**	**ON544264**	**This study**
	**SDBR-CMU412**	**ON555806**	**ON555821**	**ON544235**	**ON544250**	**ON544265**	**This study**
	**SDBR-CMU413**	**ON555807**	**ON555822**	**ON544236**	**ON544251**	**ON544266**	**This study**
	**SDBR-CMU414**	**ON555808**	**ON555823**	**ON544237**	**ON544252**	**ON544267**	**This study**
*Exophiala lavatrina*	NCPF 7893	LT594696	–	–	LT594729	–	[7]
	NCPF 7898	LT594697	–	–	LT594731	–	[7]
*Exophiala lecanii-corni*	CBS 123.33 ^T^	AY857528	FJ358311	–	–	–	[131,139]
	B2242C	MT320770	–	–	MZ190330	–	[140]
*Exophiala lignicola*	CBS:144622 ^T^	NR163358	–	–	MK442694	–	[21]
*Exophiala macquariensis*	CBS 144232 ^T^	MF619956	–	MH297438	MH297439	–	[2]
*Exophiala mali*	CBS 146791 ^T^	MW175341	–	–	–	–	[92]
*Exophiala mansonii*	CBS 101.67 ^T^	AF050247	X79318	–	–	–	[135,141]
*Exophiala mesophila*	CBS 402.95 ^T^	JF747111	JN856016	JN112476	JN128761	–	[3]
	CBS 119910	JF747113	–	JN112478	JN128753	–	[3]
*Exophiala moniliae*	CBS 520.76 ^T^	KF881967	–	–	–	–	Unpublished
	BMU00283	MW222184	–	–	–	–	Unpublished
*Exophiala nagquensis*	CGMCC 3.17333 ^T^	KP347948	KP347970	KP347924	KP347914	KP347895	[18]
	CGMCC 3.17334	KP347949	MG012741	KP347923	KP347915	KP347896	[18]
*Exophiala nidicola*	CBS 138589 ^T^	NR161045	–	–	–	–	[72]
*Exophiala nishimurae*	CBS 101538 ^T^	AY163560	KX822288	JX482552	EF551523	JX482553	[33]
*Exophiala oligosperma*	CBS 725.88 ^T^	AY163551	FJ358313	EF551508	EF551534	EF551474	[17,131]
	CBS 265.49	MH856519	–	EF551507	EF551536	EF551473	[136]
*Exophiala opportunistica*	CBS 109811 ^T^	JF747123	–	JN112486	JN128792	JN112408	[3]
*Exophiala palmae*	CMRP1196 ^T^	KY680434	–	KY689829	–	–	[107]
	CMRP1207	KY680433	–	KY689828	–	–	[107]
*Exophiala phaeomuriformis*	CBS 131.88 ^T^	AJ244259	–	–	–	–	Unpublished
*Exophiala pisciphila*	CBS 537.73 ^T^	NR121269	JN856018	JN112493	JN128788	JN112412	[3,142]
	CBS 121500	JF747134	–	JN112496	JN128789	JN112414	[3]
*Exophiala polymorpha*	CBS 138920 ^T^	KP070763	–	–	–	–	[8]
*Exophiala prostantherae*	CBS 146794 ^T^	NR171990	–	–	–	–	[92]
*Exophiala pseudooligosperma*	YMFT 1.6741	MW616557	MW616558	MZ127830	–	–	[4]
*Exophiala psychrophila*	CBS 191.87 ^T^	JF747135	JN856019	JN112497	JN128798	–	[3]
	CBS 256.92	JF747136	–	JN112498	–	–	[3]
*Exophiala quercina*	CBS 146024 ^T^	NR170053	–	–	MT223713	–	[73]
*Exophiala radicis*	P2854 ^T^	KT099204	KT723453	KT723463	KT723458	KT723443	[5]
*Exophiala salmonis*	CBS 157.67 ^T^	AF050274	JN856020	JN112499	JN128747	JN112415	[3,135]
	CBS 120274	JF747138	–	KF928562	JN128802	JN112416	[3]
*Exophiala saxicola*	**SDBR-CMU415 ^T^**	**ON555809**	**ON555824**	**ON544238**	**ON544253**	**ON544268**	**This study**
	**SDBR-CMU416**	**ON555810**	**ON555825**	**ON544239**	**ON544254**	**ON544269**	**This study**
*Exophiala siamensis*	**SDBR-CMU417 ^T^**	**ON555811**	**ON555826**	**ON544240**	**ON544255**	**ON544270**	**This study**
	**SDBR-CMU418**	**ON555812**	**ON555827**	**ON544241**	**ON544256**	**ON544271**	**This study**
*Exophiala sideris*	CBS 121818 ^T^	HQ452311	HQ441174	HQ535833	HQ452336	–	[9]
	CBS 127096	HQ452312	HQ441175	HQ535834	HQ452337	–	[9]
*Exophiala spartinae*	CBS 147266 ^T^	NR174648	–	–	–	–	[110]
*Exophiala spinifera*	CBS 899.68 ^T^	AY156976	–	EF551516	EF551541	EF551482	[33]
*Exophiala tremulae*	CBS 129355 ^T^	FJ665274	KT894147	KT894148	KT894149	KT894146	[5,89]
*Exophiala xenobiotica*	CBS 118157 ^T^	DQ182587	–	–	–	–	[27]
	CBS 117646	KP132146	–	–	–	–	[27]
*Cyphellophora eucalypti*	CBS:124764 ^T^	GQ303274	NG062860	KF928601	GU384510	JQ325009	[133,137,143,144]
*Cyphellophora fusarioides*	MUCL 44033	NR132879	NG065006	KC455224	–	–	[137]

Note: species obtained in this study are in bold. Superscript “T” indicates type species and “–” represents the absence of sequence data in GenBank.

**Table 4:** Colony diameter of 15 fungal strains on MEA at different temperatures for 28 days of incubation in the darkness.

Fungal Strains	Colony Diameter (mm) *							
	10 °C	15 °C	20 °C	25 °C	28 °C	30 °C	35 °C	37 °C
SDBR-CMU404	10.25 ± 0.27	17.92 ± 0.92	18.17 ± 0.52	23.83 ± 0.41	24.42 ± 0.58	22.83 ± 0.68	13.08 ± 0.58	8.00 ± 0.55
SDBR-CMU405	10.54 ± 0.33	17.88 ± 0.56	19.08 ± 0.12	24.27 ± 0.42	24.85 ± 0.57	23.09 ± 0.41	12.47 ± 0.52	8.12 ± 0.14
SDBR-CMU406	11.42 ± 0.52	16.12 ± 0.16	19.78 ± 0.72	24.05 ± 0.97	25.41 ± 0.44	22.79 ± 0.85	12.55 ± 0.55	7.36 ± 0.22
SDBR-CMU407	11.25 ± 0.42	16.33 ± 0.41	19.25 ± 0.52	25.58 ± 0.58	25.92 ± 0.38	23.17 ± 0.52	12.33 ± 0.41	6.92 ± 0.20
SDBR-CMU408	10.12 ± 0.22	16.45 ± 0.87	18.44 ± 0.61	25.03 ± 0.45	25.25 ± 0.62	22.81 ± 0.43	12.74 ± 0.40	7.45 ± 0.39
SDBR-CMU409	17.75 ± 0.27	20.08 ± 1.07	28.33 ± 0.98	36.24 ± 1.44	37.00 ± 1.26	40.83 ± 1.33	10.08 ± 0.38	6.08 ± 0.20
SDBR-CMU410	16.11 ± 0.18	24.35 ± 0.84	26.65 ± 0.88	35.91 ± 1.36	36.02 ± 1.31	38.42 ± 1.44	8.27 ± 0.45	5.96 ± 0.22
SDBR-CMU411	14.98 ± 0.12	20.03 ± 0.41	27.78 ± 1.23	34.78 ± 0.97	35.43 ± 1.28	36.92 ± 1.96	9.04 ± 0.36	5.23 ± 0.27
SDBR-CMU412	15.97 ± 0.52	26.27 ± 0.92	27.56 ± 0.71	36.77 ± 1.22	37.11 ± 1.45	38.82 ± 0.79	9.19 ± 0.24	5.71 ± 0.13
SDBR-CMU413	14.42 ± 0.38	19.25 ± 0.27	25.08 ± 1.07	32.25 ± 1.44	34.33 ± 2.04	35.42 ± 0.86	8.42 ± 0.49	5.58 ± 0.49
SDBR-CMU414	14.23 ± 0.47	25.78 ± 0.74	25.71 ± 0.88	35.04 ± 1.47	35.47 ± 1.42	36.96 ± 0.65	10.12 ± 0.56	5.44 ± 0.39
SDBR-CMU415	9.92 ± 0.20	14.42 ± 0.49	15.50 ± 0.55	21.33 ± 0.26	23.75 ± 1.37	24.17 ± 1.66	12.75 ± 0.27	8.92 ± 0.20
SDBR-CMU416	9.83 ± 0.41	14.08 ± 0.20	16.58 ± 0.38	21.75 ± 1.13	24.67 ± 0.41	26.88 ± 1.28	11.42 ± 0.20	8.17 ± 0.26
SDBR-CMU417	8.08 ± 0.38	10.08 ± 0.49	11.75 ± 1.17	10.42 ± 0.80	9.42 ± 0.58	7.33 ± 0.26	–	–
SDBR-CMU418	8.08 ± 0.38	10.50 ± 0.77	11.83 ± 0.68	10.35 ± 1.17	9.92 ± 0.20	7.58 ± 0.20	–	–

* The results are mean ± standard deviation and “–” represents no growth.

## Data Availability

The DNA sequence data obtained from this study have been deposited in GenBank under the accession numbers; ITS (ON555798, ON555799, ON555800, ON555801, ON555802, ON555803, ON555804, ON555805, ON555806, ON555807, ON555808, ON555809, ON555810, ON555811, ON555812); nrSSU (ON555813, ON555814, ON555815, ON555816, ON555817, ON555818, ON555819, ON555820, ON555821, ON555822, ON555823, ON555824, ON555825, ON555826, ON555827); *tub* (ON544227, ON544228, ON544229, ON544230, ON544231, ON544232, ON544233, ON544234, ON544235, ON544236, ON544237, ON544238, ON544239, ON544240, ON544241); *tef* (ON544242, ON544243, ON544244, ON544245, ON544246, ON544247, ON544248, ON544249, ON544250, ON544251, ON544252, ON544253, ON544254, ON544255, ON544256) and *act* (ON544257, ON544258, ON544259, ON544260, ON544261, ON544262, ON544263, ON544264, ON544265, ON544266, ON544267, ON544268, ON544269, ON544270, ON544271).
